# Down-regulated of SREBP-1 in circulating leukocyte is a risk factor for atherosclerosis: a case control study

**DOI:** 10.1186/s12944-019-1125-1

**Published:** 2019-10-14

**Authors:** Chunyan Peng, Pan Lei, Xiandong Li, Huaqiang Xie, Xiaowen Yang, Tao Zhang, Zheng Cao, Jicai Zhang

**Affiliations:** 1 Department of Laboratory Medicine, Taihe hospital, Hubei University of Medicine, Shiyan, China; 2 Department of Cardiology, Taihe hospital, Hubei University of Medicine, Shiyan, China; 3 Department of Neurosurgery, Taihe hospital, Hubei University of Medicine, Shiyan, China

**Keywords:** SREBP, Coronary atherosclerotic disease, Dyslipidemia, Carotid plaques, miR-33

## Abstract

**Background:**

Sterol regulatory-element binding proteins (SREBPs) and mir-33 (miR-33a, miR-33b), which are encoded by the introns of SREBPs, are key factors in the lipid metabolism pathway. SREBPs mRNA in circulating leucocyte and carotid plaques, along with various risk factors that associated with Coronary Atherosclerotic Disease (CAD) were investigated in a central Chinese cohort.

**Methods:**

A total of 218 coronary atherosclerotic disease (CAD) patients, and 178 non-CAD controls, were recruited to collect leukocytes. Carotid plaques and peripheral blood were obtained from CAD patients undergoing carotid endarterectomy (CEA) (*n* = 12) while THP-1 and peripheral blood mononuclear cells (PBMCs) were stimulated with Oxidized low-density lipoprotein (ox-LDL) to establish an in vitro foam cell formation model. SREBPs and miR-33 levels were quantified by qPCR. Routine biochemical markers were measured using standard procedures.

**Results:**

SREBP-1 mRNA level of circulating leucocytes in CAD patients were significantly lower than in non-CAD controls (*p* = 0.005). After stratification coronary artery atherosclerotic complexity, we detected a significant reduction of SREBP-1 in high-risk complexity CAD patients (SYNTAX score > 23) (*p* = 0.001). Logistic regression analysis indicated that decreased expression of SREBP-1 was a risk factor of CAD (odds ratio (OR) =0.48, 95% confidence interval (CI) = 0.30~0.76, *p* = 0.002) after adjusting clinical confounders; the mRNA levels of SREBPs in carotid plaques correlated with the corresponding value in circulating leukocytes (SREBP-1 r = 0.717, *p* = 0.010; SREBP-2 r = 0.612, *p* = 0.034). Finally, there was no significant difference in serum miR-33 levels between CAD patients and controls.

**Conclusions:**

Our finding suggesting a potential role in the adjustment of established CAD risk. The future clarification of how SREBP-1 influence the pathogenesis of CAD might pave the way for the development of novel therapeutic methods.

## Background

Coronary atherosclerotic disease (CAD) are responsible for one-third of all deaths worldwide. Dyslipidemia, a condition which involves elevated levels of cholesterol and triglyceride, but reduced levels of high-density lipoprotein (HDL), has been reported to be correlated with over half of the cases of CAD reported globally. It is estimated that the number of people with dyslipidemia will reach 78 million by the year 2022 [1], which could impose a substantial medical burden not only to individuals but also to the whole society. Membrane-bound, basic helix-loop-helix leucine zipper (bHLH-LZ) transcription factors, referred to as sterol regulatory element-binding proteins (SREBPs), play a central role in regulating genes that are important for the biosynthesis and uptake of lipids [2, 3]. The two mammalian SREBP genes, SREBF1 and SREBF2, have distinct but overlapping lipogenic transcriptional programs. Generally, SREBP-1 activates the synthesis of fatty acids and triglycerides while SREBP-2 increases the synthesis of cholesterol [2]. All SREBPs are synthesized as transcriptionally inactive precursors that are bound to the endoplasmic reticulum (ER) and the nuclear envelope. Their transcriptional activation requires binding to SREBP cleavage-activating protein (SCAP); binding translocate their inactive precursors form the ER to the Golgi and subsequent migration into nucleus, where they can bind to sterol response elements (SREs) in the promoter region of target genes and thereby modulate gene transcription [4]. Moreover, activation of SREBP transcription leads to the increased expression of microRNAs-33, miR-33a and miR-33b, which are located within intron 16 of SREBP-2 and intron 17 of SREBP-1, respectively [5]. The co-expression of the two miR-33 forms, along with their host genes, can function in a synergistic manner to further facilitate a return to homeostasis [6].

Over recent years, a multitude of in vivo and in vitro studies have demonstrated that SREBPs can integrate multiple forms of cell signals in order to control lipogenesis, as well playing roles in a range of unexpected pathways associated with a variety of diseases, including diabetes mellitus, nonalcoholic fatty liver disease, chronic kidney disease, dyslipidemia, cardiac arrhythmias and obesity [3, 7, 8]. However, we know very little about the association between SREBPs, CAD and atherogenic risk factors that are prevalent in the Chinese population. The objective of our study was to evaluate the relationship between circulating leukocyte SREBPs, carotid plaques levels and CAD, and compare the data with both established and emerging circulating biomarkers for cardiac risk in the Chinese population.

## Methods

### Participants

This case-controlled study was performed in accordance with the ethical guidelines of the Declaration of Helsinki and was approved by the Medical Ethics Committee of Taihe Hospital of Hubei University of Medicine. Written and informed consent was obtained from all participants prior to entering the study. The designment of the study was illustrated in the flow chart Additional file 1: Figure S1. A total of 218 patients with CAD, 12 CAD patients with symptomatic carotid atherosclerotic diseases who were scheduled for carotid endarterectomy (CEA) and 178 healthy control subjects, were recruited in Taihe Hospital, Hubei University of Medicine, Hubei, China, from August 2016 to January 2019. The inclusion criteria were the same as those described in our previous reports [9]. CAD was confirmed in all patients by angiography; Comorbidities and medication were assessed by self-reported information and written medical reports. Patients were excluded if they had acute heart failure. Age-matched and gender-matched healthy individuals, with a normal angiogram, were selected as controls (the non-CAD group). All control participants showed no signs of cardiac or systemic diseases based on physical examination records at enrollment. Hypertension was determined as a systolic blood pressure ≥ 140 mmHg, and/or a diastolic blood pressure ≥ 90 mmHg and/or the use of anti-hypertensive medication. Diabetes mellitus was defined as fasting plasma glucose levels ≥7.0 mmol/L, and/or post-load plasma glucose levels ≥11.1 mmol/L, and/or self-reported use of anti-diabetic medication. Hyperlipidemia was defined as a fasting serum TG (triglycerides) > 1.7 mmol/L, and/or TC (total cholesterol) > 5.72 mmol/L, and/or HLD-C (high-density lipoprotein cholesterol) < 0.91 mmol/L, and/or LDL-C (low-density lipoprotein cholesterol) > 3.64 mmol/L, and/or taking therapeutic lipid-lowering medication. Other relevant data were collected from participants by interviews or from medical case files or self-administered questionnaires.

### Peripheral blood and carotid plaque preparation

At baseline, peripheral blood samples were collected after fasting for at least 8 h. Samples from emergency CAD patients were collected immediately after hospitalization. Following collection, samples were allowed to clot for 1 h at room temperature and then centrifuged for 15 mins at 1000 g. Serum samples were stored at − 80 °C until analysis. Carotid plaques, and adjacent carotid tissue, were collected during carotid endarterectomy (CEA) operations and banked in liquid nitrogen for < 3 months prior to testing. Cryoprotectant solution (a final concentration of 10% Dimethyl sulfoxide (DMSO)) was used to prevent ice crystals damaging the tissue, as described elsewhere [10].

### Laboratory detection panel

A panel of 15 biomarkers that have previously been associated with CAD were selected and analyzed in baseline blood samples. The selected biomarkers have proposed links with atherosclerosis through a variety of different pathological mechanisms: inflammatory markers (high-sensitivity C-reactive protein (hs-CRP)); lipid-metabolic markers (TC, TG, HDL-C, LDL-C, apolipoprotein A1 (ApoA1), apolipoprotein B (ApoB), lipoprotein a (LP(a))); markers of myocardial enzymes (aspartate aminotransferase (AST), creatine kinase-MB (CK-MB), lactate dehydrogenase (LDH), hydroxybutyrate dehydrogenase (HBDH); protein-metabolic marker (homocysteine (HCY)); marker of renal function (cystatin-C (Cys-C) and a glucose-metabolic marker (glycated serum protein (GSP)). Serum/plasma samples were stored at − 80 °C prior to assays and were thawed only once prior to use. All biochemical parameters were detected by standard techniques, which were performed in the Core Laboratory of Taihe Hospital, Hubei University of Medicine.

### Assessment and categorization of SYNTAX score

Coronary angiograms were analyzed by 2 experienced interventional cardiologists who were blinded for the analyzed variables except for age and gender. Each lesion with >The SYNTAX score was calculated using the internet-based SYNTAX calculator version 2.28 (www.syntaxscore.com). In line with previous studies investigating the impact of different parameters on CAD complexity, we used a cut off SYNTAX score of < 23 versus ≥23 according to low versus high risk complexity of CAD [11–13].

### Cell culture and treatment

Human monocytic THP-1 cells were cultured in RPMI-1640 medium (Gibco, USA) containing 10% fetal bovine serum (FBS) (Gibco, USA) and incubated with 50 ng/mL of PMA for 48 h to differentiate into macrophages. PBMCs were isolated from the heparinized blood of healthy donors by centrifugation through a Ficoll/Hypaque solution (14.077 g/ml, Sigma Chemical Co., Munich, Germany). PBMCs were then plated into gelatin flasks at 37 °C for 45mins to collect monocytes. After detachment with 10 mM of EDTA, monocytes were washed with DMEM and resuspended in DMEM containing 10% FBS, 100 μg/L of streptomycin, 100 IU of penicillin, and 1% nonessential amino acids, 2 mM of glutamine, and plated in 48-well plates (2.5 * 10^5^ cells/well). Monocytes differentiated into macrophages during in vitro culture for 7 days [14]. Subsequently, THP-1 and PBMC macrophages were exposed to Ox-LDL in 2 ml of RPMI-1640 culture growth medium containing 10% FCS for 48 h. To determine intracellular lipid contents, cells were removed from the culture plates and washed twice with PBS. Then, intracellular lipids were extracted using isopropanol. Total and free cholesterol were determined using an enzymatic assay.

### RNA extraction and quantification real-time PCR

Total RNA was extracted form leukocytes, atherosclerotic plaques and cultured cells using TRIZOL Regent (Invitrogen, Carlsbad, CA) and cDNA synthesis was performed with a HiScript® 1st strand cDNA Synthesis Kit (Nanjing Vazyme Biotech Co., Ltd., Nanjing, China). Five nanograms of cDNA were used for real-time reactions using ChamQTM SYBR® qPCR Master Mix (Nanjing Vazyme Biotech Co., Ltd., Nanjing, China) and a ViiATM 7 Real-time PCR System (Applied Biosystems). Relative gene expression levels were calculated using the comparative crossing threshold method of relative quantification (ΔCq) and fold change (FC) values. ΔCq was designated as the mean Cq (mean of duplicates) of a target gene subtracted by the mean Cq (mean of duplicates) of a reference gene (GAPDH). Based on recommendations from the manufacturer, the Cq expression cut-off was set to 30; this was applied to all calculations. In order to compare mean expression levels between the CAD group and the control group, FC was designated as 2-ΔCq. Detailed primer information and qPCR data processing are shown in Additional file 1: Table S1.

### Statistical analysis

The power and sample size calculated by R (R version 3.4.2) (Additional file 1: Figure S2). The Continuous variables are expressed as mean ± standard deviation (SD) or as median (inter-quartile range), as appropriate, based on distributions. Continuous variables were compared by the student’s t test or Mann-Whitney U test and categorical variables by the chi-squared test. A paired-sample t test used to compare expression values between carotid plaques and circulating leukocytes in the 12 patients who underwent CEA. A paired-sample correlation was used to explore expression correlation between carotid plaques and circulating leukocytes. Additional correlation analysis was performed using the nonparametric Spearman’s rank correlation test. Conditional logistic regression analyses were performed to assess whether reduced values of SREBP-1 expression correlated independently with traditional atherosclerosis/CAD risk factors. All statistical significance levels were set at *p* < 0.05 (two-sided) and all statistical analyses were performed using SPSS version 23.0 (SPSS Inc., Chicago, IL, USA).

## Results

### Baseline characteristics

The principal characteristics of all subjects are summarized in Table 1. There were no statistically significant differences observed in terms of age, gender, LDH, CysC or GSP when compared between the control and CAD groups (*p* = 0.214, 0.120, 0.272, 0.445, 0.086, respectively). In terms of lipid parameters, levels of TG and LP(a) were significantly higher in CAD patients than in controls, while levels of TC, HDL-C, LDL-C, APO-A1 and APB-B were significantly reduced in CAD patients (all *p* < 0.001). Compared to the control group, CAD patients showed a more unfavorable inflammatory reaction; CAD patients had higher levels of hs-CRP (*p* < 0.001) and higher levels of the myocardial enzymes AST (*p* = 0.003), CK-MB (*p* < 0.001) and HBDH (*p* = 0.042).

**Table 1:** Baseline and clinical characteristics of the participants

Characteristic	Controls(*n* = 178)	CAD patients(*n* = 218)	*p* value
Demographics			
Age (years)	57.74 ± 8.48	58.98 ± 9.32	0.214
Male	116	141	0.120
Risk factors			
Hypertension (%)	6	125	< 0.001
Diabetes mellitus (%)	2	46	< 0.001
hyperlipidemia (%)	0	32	< 0.001
Clinical parameters			
TC (mmol/L)	4.81 ± 0.94	3.75 ± 1.06	< 0.001
TG (mmol/L)	1.26 (0.82, 1.48)	1.59 (0.79, 2.04)	< 0.001
HDL-C (mmol/L)	1.41 ± 0.33	1.01 ± 0.26	< 0.001
LDL-C (mmol/L)	2.27 ± 0.586	1.91 ± 0.70	< 0.001
Apo-A1 (g/L)	1.62 ± 0.21	1.48 ± 0.28	< 0.001
Apo-B (g/L)	0.91 ± 0.20	0.82 ± 0.24	< 0.001
LP(a) (mg/L)	78.20(16.79, 120.33)	115.84(33.29,184.10)	< 0.001
AST (IU/L)	25.14 (20.00, 29.17)	30 (17.75,35.25)	0.003
CK-MB (IU/L)	8.43 ± 6.90	15.85 ± 101.90	< 0.001
LDH (IU/L)	187.43 ± 42.60	193.49 ± 66.17	0.272
HBDH (IU/L)	119.69 ± 30.05	128.19 ± 51.66	0.042
hs-CRP (mg/L)	5.26 (2.17, 5.71)	7.00 (4.75, 12.33)	< 0.001
HCY (μmol/L)	11.30 (8.90,12.50)	11.2 (8.07, 13.40)	0.012
CysC (mg/L)	0.59 ± 0.14	0.60 ± 0.21	0.445
GSP (mmol/L)	2.61(2.46, 2.74)	2.62(2.39, 2.89)	0.086
SREBP-1 (fold change)	1.00 ± 1.17	0.66 ± 1.14	0.005
SREBP-2 (fold change)	1.00 ± 0.90	0.93 ± 1.34	0.528
Statins Medication	0	69	< 0.001

### Differentially expressed SREBP mRNA in CAD patients

Compared with the control group, the CAD group had significantly lower levels of SREBP-1 mRNA; overall, these levels showed a reduction of approximately 35% (*p* = 0.005) (Table 1). In contrast, SREBP-2 mRNA levels showed no significant difference between the two groups (*p* = 0.528).

To further investigate whether the observed reductions in SREBP were associated with disease severity, we analyzed SREBP expression variation in low- (SYNTAX score of < 23) and high-risk complexity patients. As shown in Table 2, compared to non-CAD subjects, the levels of SREBP-1 mRNA were significantly down-regulated (by almost 50%) in high-risk CAD patients (*p* = 0.001), although this difference was abolished in low-risk patients (*p* = 0.357). In contrast, SREBP-2 mRNA did not differ significantly when compared across the two risk groups.

**Table 2:** Clinical parameters and SREBPs expression in low-risk complexity, high-risk complexity and non-CAD control participants

Characteristic	CAD patients		Non-CAD Controls *n* = 178	*P* value	
	GROUP A^1^ *n* = 77	Group B ^2^ *n* = 141		*P* ^3^	*P* ^4^
Demographics					
Age (years)	58.33 ± 9.28	59.32 ± 9.35	58.98 ± 9.32	0.617	0.114
Male	45	96	141		
Clinical parameters					
TC (mmol/L)	3.94 ± 1.01	3.64 ± 1.06	4.81 ± 0.94	< 0.001	< 0.001
TG (mmol/L)	1.32(0.88, 2.03)	1.15(0.69, 2.06)	1.26(0.82, 1.48)	0.004	0.335
HDL-C (mmol/L)	1.08 ± 0.26	0.97 ± 0.25	1.41 ± 0.33	< 0.001	< 0.001
LDL-C (mmol/L)	1.97 ± 0.68	1.87 ± 0.71	2.27 ± 0.59	< 0.001	< 0.001
Apo-A1 (g/L)	1.57 ± 0.26	1.43 ± 0.29	1.62 ± 0.21	0.157	< 0.001
Apo-B (g/L)	0.82 ± 0.22	0.82 ± 0.25	0.91 ± 0.21	0.002	< 0.001
LP(a) (mg/L)	102.8 ± 92.61	123.70 ± 110.20	78.20 ± 84.93	0.048	< 0.001
AST (IU/L)	23.0(16.5, 29.0)	23.00(18.00, 37.00)	25.14(20.00, 29.17)	0.314	0.488
CK-MB (IU/L)	16.66 ± 10.10	15.38 ± 10.27	8.43 ± 6.90	< 0.001	< 0.001
LDH (IU/L)	190.13 ± 47.62	195.52 ± 74.70	187.43 ± 42.60	0.655	0.783
HBDH (IU/L)	124.81 ± 37.79	130.14 ± 58.08	119.69 ± 30.05	0.250	0.050
hs-CRP (mg/L)	6.13(4.51, 8.27)	8.05(4.81, 14.90)	5.26(2.17, 5.71)	< 0.001	< 0.001
HCY (μmol/L)	9.90(7.50,12.45)	11.75(8.65, 13.68)	11.30(8.90,12.50)	0.190	0.007
CysC (mg/L)	0.59 ± 0.21	0.61 ± 0.22	0.59 ± 0.14	0.882	0.323
GSP (mmol/L)	2.70(2.43, 2.94)	2.57(2.37, 2.86)	2.61(2.46, 2.74)	0.089	0.856
SREBP-1 (fold change)	0.85 ± 1.15	0.56 ± 1.12	1.00 ± 1.17	0.357	0.001
SREBP-2 (fold change)	1.12 ± 1.29	0.83 ± 1.39	1.00 ± 0.89	0.450	0.206

^1^ Group A, SYNTAX score of < 23; ^2^ Group B, SYNTAX score of ≥23; ^3^ Group A compare with non-CAD controls; ^4^ Group B compared with non-CAD controls

### Associations between SREBPs and risk factors for CAD

The association between lower levels of SREBP-1 mRNA and an increased risk for CAD was further evaluated by logistic stepwise regression. As summarized in Table 3, odds ratios (ORs) were significant after adjustment for age, gender (OR = 0.77, 95% confidence interval [CI] = 0.64~0.93, *p* = 0.006), although these associations were abolished when history of hyperlipidemia, diabetes and hypertension was included as covariates (OR = 0.84, 95%CI = 0.64~1.11, *p* = 0.226), the decreased SREBP-1 expression was still a significant risk factor for CAD after adding Statins medication as a covariate (OR = 0.48, 95% CI 0.30~0.76, *p* = 0.002).

**Table 3:** Conditional logistic regression analysis of independent correlates between circulating leukocyte SREBPs mRNA variability and CAD

		Crude model	Established risk factors 1	Established risk factors2
SREBP-1	Odds ratio	0.77	0.84	0.48
	95% CI	0.64~0.93	0.64~1.11	0.30~0.76
	*p*-value	0.006	0.226	0.002
SREBP-2	Odds ratio	1.08	1.05	1.43
	95% CI	0.88~1.32	0.80~1.38	1.03~2.00
	p-value	0.434	0.728	0.032

This crude model was adjusted for age and gender; established risk factor 1 was adjusted for age, gender, hyperlipidemia, diabetes and hypertension; established risk factor 2 was adjusted for age, gender, hyperlipidemia, diabetes, hypertension and medication of Statins.

### Correlation between SREBP mRNA expression and risk factors for CAD

To evaluate whether deterministic risk factors of CAD are correlated with SREBP expression, we performed correlation analysis for both non-CAD controls and CAD patients. As described in Additional file 1: Table S2, according to the Pearson or Spearman correlation method, leukocyte SREBP-1 shows a positive association with TC (r = 0.257, *p* = 0.001), LDL (r = 0.162, *p* = 0.030), Apo-A1(r = 0.296, *p* < 0.001), Apo-B (r = 0.232, *p* = 0.002) and CysC (r = 0.380, *p* < 0.001) but shows negative association with CK-MB (r = − 0.284, *p* < 0.001), hs-CRP (r = − 0.284, *p* < 0.001) in non-CAD controls. In the CAD group, SREBP-1 showed positive associations with HDL (r = 0.171, *p* = 0.011), Apo-A1(r = 0.145, *p* = 0.033) and CysC (r = 0.150, *p* = 0.027), GSP (r = 0.229, *p* = 0.001) and negative associations with CK-MB (r = − 0.201, *p* = 0.003), LDH (r = − 0.146, *p* = 0.031) and hs-CRP (r = − 0.284, *p* < 0.001) in non-CAD controls. In contrast, there was no significant correlation between SREBP-2 and any estimating risk factors mentioned above (Additional file 1: Table S2). In addition, a significant correlation was observed between SREBP-1 and SREBP-2 in the control group (r = 0.373, *p* < 0.001) and the CAD group (r = 0.512, *p* < 0.001).

### The expression of SREBPs in carotid plaques

**Fig. 1:**
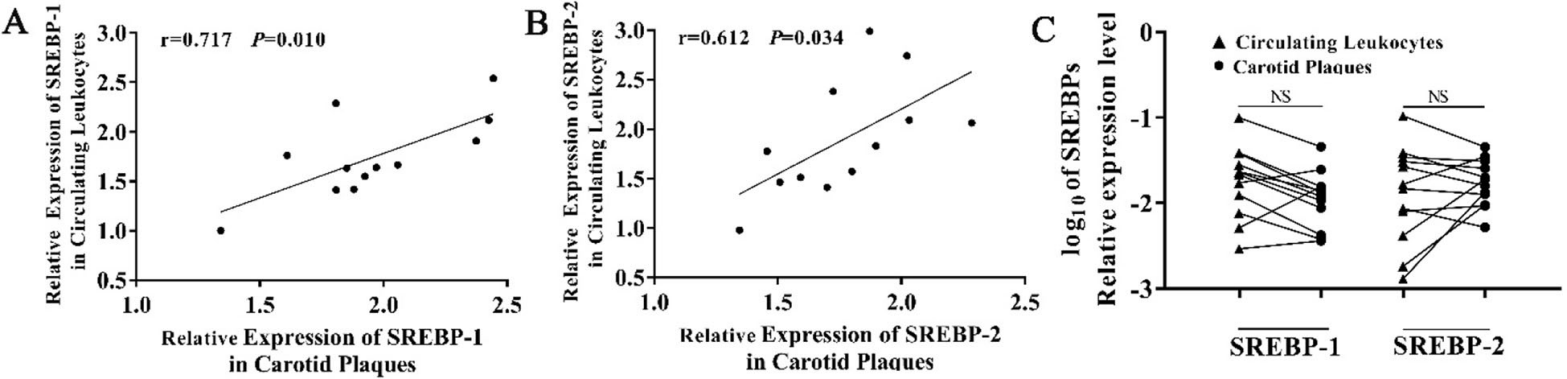
SREBP expression in carotid plaques and circulating leukocytes. **a**, Correlation of SREBP-1 expression in circulating leukocytes and carotid plaques; **b**, Correlation of SREBP-2 expression in circulating leukocytes and carotid plaques; **c**, Comparison of SREBP-1 and SREBP-2 expression in carotid plaques and circulating leukocytes in the 12 CAD participants. NS: not significant change

Carotid and coronary artery disease are two major atherosclerotic conditions that are closely related [15, 16]. In this study, we collected 12 carotid plaques as surrogates for foam cells to explore variation in SREBP expression (Additional file 1: Figure S3). As illustrated in Fig. 1a and Fig. 1b, the mRNA levels of both SREBP-1 and SREBP-2 in carotid plaques were closely correlated with their corresponding values in circulating leukocytes (SREBP-1 r = 0.717, *p* = 0.010; SREBP-2 r = 0.612, *p* = 0.034). However, there were no significant differences when compared between carotid plaques and circulating leukocytes (Fig. 1c) (*p* > 0.05).

### The expression of SREBPs in foam cells

**Fig. 2:**
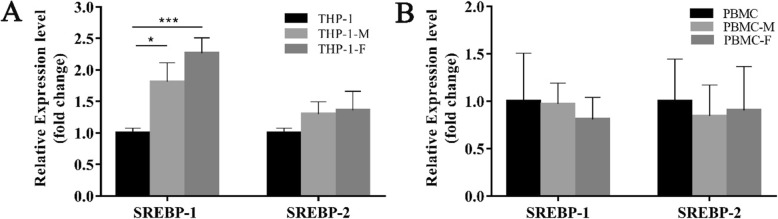
SREBPs mRNA expression in ox-LDL-induced foam cells. **a**, SREBPs mRNA levels in THP-1 monocytes, THP-1 macrophages, ox-LDL-induced THP-1 foam cells. **b**, SREBPs mRNA levels in PBMC monocytes, PBMC macrophages, ox-LDL-induced PBMC foam cells. THP-1, THP-1 monocytes; THP-1-M, THP-1 macrophages; THP-F, THP-1 foam cells; PBMC, Peripheral blood mononuclear cells; PBMC-M, PBMC macrophages; PBMC-F, ox-LDL-induced PBMC foam cells, *** *p* = 0.001, * *p* = 0.011

To further determine the role of SREBPs in the process of foam cell formation, we examined the mRNA expression of SREBPs in foam cells derived from THP-1 cells and PBMC. As shown in Fig. 2., compared to the control group (THP-1 monocytes and PBMC monocytes), levels of SREBP-1 mRNA were significantly increased in Ox-LDL-induced THP-1 foam cells (*p* < 0.05, THP-1 foam cells vs THP-1 monocytes). However, there was no significant variation regarding foam cells derived from PBMCs. Consistent with our findings in circulating leukocytes, SREBP-2 mRNA expression levels showed no significant variation during THP-1 and PBMC-derived foam cell formation.

### miR-33 and risk factors for CAD

The principal characteristics of the subjects which were used to detect miR-33 are summarized in Additional file 1: Table S3. Data show that levels of plasma miR-33a and miR-33b were not significantly different in CAD patients (*n* = 67) when compared to age- and gender-matched controls (*n* = 73). To evaluate whether clinical risk factors of CAD correlated with miR-33, we performed a correlation analysis across all participants. As described in Additional file 1: Table S4, according to the Pearson/Spearman correlation method, plasma miR-33a showed positive associations with TG (r = 0.169, *p* = 0.049), AST (r = 0.217, *p* = 0.011) and GSP (r = 0.240, *p* < 0.005). However, no significant correlation was observed between miR-33b and any of the risk factors (all *p* > 0.05). Furthermore, there was no significant association between circulating leukocyte SREBPs mRNA and plasma mir-33 (all *p* > 0.05).

## Discussion

To the best of our knowledge, this is the first study to investigate the expression variation of SREBPs in circulating leukocytes in patients with CAD. Our data generated four main findings. First, we found that CAD patients had higher levels of serum TG content but lower levels of SREBP-1 mRNA in leukocytes. Transcriptionally, SREBP-1 mRNA is strongly induced by insulin via a mechanism involving the liver X receptor (LXR) transcription factors, as well as by SREBP-1 feed-forward activation [11, 17]. Translocation of the SREBP-N terminal is a highly regulated process which occurs via activation of the PI3K/AKT/mTROC1 pathway. The mature, translocation-active form of SREBP protein is regulated by glycogen synthase kinase-3β (GSK-3β) inhibition [18] and Lipin1 phosphorylation [19]. As an important activator in the transcriptional regulation of genes involved in fatty acid and TG metabolism, the down-regulation of SREBP-1 might be associated with serum TG disturbance; it is possible that the accumulation of lipids blocked the translocation and cleavage of the SREBP-SCAP complex and disrupts lipid homeostasis, a process based upon self-regulation feedback. Moreover, decreased SREBP-1 was a risk factor for CAD, independent of histories of hyperlipidemia, diabetes, hypertension and medication of Statins, which suggest SREBP-1 might be involved in the pathogenesis of CAD through unconventional regulatory network which still need to be clarified with large sample size in future.

Secondly, CAD patients showed a significant reduction in serum TC, LDL-C, Apo-A1, Apo-B and GSP levels; however, there were no significant changes in SREBP-2 mRNA expression. This pattern of lipid variation was similar with findings related to atherogenic dyslipidemia in our previous studies [9, 20], which is quite different from what has been reported in other populations; generally, CAD patients in other studies showed an increased level of blood cholesterol. We speculate that this discrepancy may be partly related to the different races or ethnic groups involved, and more importantly, the medications of the participants. The majority of the CAD patients involved in the current study were exposed to related primary disorders prior to recruitment and over 30% of the patients were taking statins as cholesterol-reducing medication on a daily basis (Additional file 1: Table S5). SREBP-2 is considered to be closely involved in the regulation of cholesterol metabolism and is controlled by intracellular cholesterol level. Statin medication, which inhibits HMG-CoA reductase (HMGCR) and cholesterol synthesis, is thought to activate SREBP-2 by reducing the amount of cholesterol in the regulatory pool of the cell. Levels of circulating leukocyte SREBP-2 in CAD patients were not significantly different from non-CAD patients in our study. This may have arisen due to the therapeutic effects of statin treatment. It is also possible that this was the reason that the average serum cholesterol level of CAD patients was controlled at a relatively low level within the normal reference range.

Thirdly, for a single individual, the levels of SREBPs expression in carotid plaques was closely related with the corresponding value in circulating leukocytes. Carotid and coronary artery disease are two major atherosclerotic conditions that share similar characteristics and mechanisms irrespective of the location site [21]. Coronary lesions were detected in almost one-third of patients who underwent coronary angiography before CEA [22]. Since we were not able to gather the tissue in coronary artery plaque lesions in CAD patients, we collected 12 carotid plaques from 12 CAD patients who underwent CEA as surrogates to study the SREBP expression in plaques. While the down-regulation of SREBP-1 in circulating leukocytes was related with CAD in this population-based study, the expression level of SREBPs in carotid plaques showed no difference to the levels in circulating leukocytes, which might suggested that it is the circulating leucocytes with aberrant SREBP expression accumulating in impaired vascular endothelial cells that build up the fundamental structure of plaque and blood samples could possibly be used as surrogates for plaques and provide prognostic information relating to atherosclerotic disease. However, this still need to be verified in large sample datasets and evaluated after adjustment for other confounders.

Finally, it is worth noting that after treatment with ox-LDL, the SREBP-1 level was increased significantly during the progression of THP-1 foam cell formation, which is in stark contrast to the decreasing trend in the circulating cells of CAD patients. Consistent with our finding, Varghese et al. [23] also showed that ox-LDL increased the expression of SREBP-1, and its downstream proteins, at both RNA and protein levels in U937 monocytes and monocyte-derived macrophages, they also demonstrate that SREBP-1 could be a key player in ox-LDL-induced excessive lipid accumulation leading to macrophage foam cell formation via the ROS-mediated NLRP3/IL-1β/SREBP-1 pathway. The discrepancy in SREBP-1 mRNA expression between the circulating leukocytes and in vitro THP-1 monocytes in our research may be due to the heterogeneous composition of different blood cells within whole blood samples. In the peripheral blood cells, monocytes account for less than 10% of the total cell counts, while circulating immune cells (lymphocytes), inflammatory cells (granulocytes), clotting factor (platelets), and other plasma components such as exosome-transported non-coding RNA, collectively build up a complicated microenvironment to influence the expression of SREBP-1. These inconsistent findings indicate that different leukocyte subtypes might have different weight in the regulatory network in the lipid homeostasis and further in vitro studies based on sorting cells are necessary.

Furthermore, previous reports showed that loss of miR-33 in low-density lipoprotein receptor null (LDLR−/−) mice led to an increased in plasma HDL-cholesterol levels and promoted reverse cholesterol transport; other studies have shown that the genetic ablation of miR-33 in ApoE ^−/−^ mice markedly reduced the progression of atherosclerosis. However, in the present study, there was only a weak correlation observed between miR-33a and TG levels. We speculated that this may be due to the limited detectability of miR-33 in the serum. Although we recruited over 400 participants in total, less than half of the serum samples tested showed a reliable detection of miR-33 expression by qPCR. This raised the question of whether circulating miR-33 could represent a dependable diagnostic biomarker or therapeutic target for metabolic-related disorders. In the future it might be necessary to use enriched exosomes to detect circulating miR-33 levels so as to fully evaluate the reliability of its diagnostic value.

To date, this represents the first study to explore the variation in expression of SREBPs in CAD. Our work contributes to a better understanding of the role of SREBPs and might lead to new avenues with which to improve therapeutic options and disease management strategies. Nevertheless, the present study still has several limitations which need to be considered. First, our study is limited by the unknown generalizability to other age and ethnic groups; additional large-scale studies in other ethnicities and in other age groups are needed to properly validate our findings. Second, we also need to detect the levels of circulating SREBP proteins, which play a key lipid-regulatory role; occasionally, these is an inconsistency between translation and transcription efficiency lead by complications in the regulatory network. Thirdly, the majority of the CAD patients involved in this study were exposed to related primary disorders prior to recruitment and some of them received a very diverse medication or nutraceuticals on a daily basis which might have important impacts on the lipid homeostasis of individuals [24]; additional precise stratification by the therapeutic regimen on larger sample size are needed to elucidate the mechanism. Furthermore, this study cannot confirm the relationships between cause and effect; the reduced levels of SREBP-1 mRNA may be merely a marker for risk, or a compensatory mechanism, and thus warrants further investigation.

## Conclusions

SREBP-1 mRNA expression levels in circulating leukocytes were significantly different in CAD patients compared to normal controls, suggesting a potential role for such data to be used in the adjustment of established CAD risk. The future clinical application of our findings, and the underlying mechanisms responsible for how SREBP-1 co-works with established risk factors to influence CAD have yet to be determined.

## Supplementary information

**Additional file 1: Table S1.** Primer sequences used for qPCR. **Table S2.** Pearson/Spearman correlation coefficients between circulating leukocyte SREBP-1, SREBP-2 and CAD risk factors in controls. **Table S3.** Baseline and clinical characteristics of the participants. **Table S4.** Pearson/Spearman correlation coefficients between plasma mir-33 and CAD risk factors in all participants tested for miR-33. **Table S5.** Clinical characteristics and SREBP mRNA in CAD with different chronic pathological conditions. **Figure S1.** The flow charts of the study. A, flow chart of case-control trials; B, flow chart of the in vitro study. **Figure S2.** The flow charts of sample size calculation. **Figure S3.** 12 Carotid plaques from CAD patients undergoing carotid endarterectomy.

## Data Availability

The authors confirm that all materials described in the manuscript are fully available to any scientist wishing to use them, without restriction.

## Abbreviations

Apo-A1: Apolipoprotein A1
Apo-B: Apolipoprotein B
AST: Aspartate aminotransferase
bHLH-LZ: Basic helix-loop-helix leucine zipper
CAD: Coronary artery disease
CAD: Coronary atherosclerotic heart disease
cDNA: Complementary deoxyribonucleic acid
CED: Carotid endarterectomy
CK-MB: Creatine kinase
CysC: Cystatin C
DMSO: Dimethyl sulfoxide
ER: Endoplasmic reticulum
FBS: Fetal bovine serum
GSP: Glycated serum protein
HBDH: Hydroxybutyrate dehydrogenase
HCY: Homocysteine
HDL-C: High-density lipoprotein cholesterol
hs-CRP: High-sensitivity C-reactive protein
LDH: Lactate dehydrogenase
LDL-C: Low-density lipoprotein cholesterol
LP(a): Lipoprotein a
mRNA: Message ribonucleic acid
PBMC: Peripheral blood mononuclear cell
PBS: Phosphate butter solution
SCAP: SREBP cleavage-activating protein
SREBP: Sterol regulatory element binding proteins
SREs: Sterol response elements
TC: Total cholesterol
TG: Triglycerides
