# Supplementary material for: Down-regulated of SREBP-1 in circulating leukocyte is a risk factor for atherosclerosis: a case control study
Source: Lipids Health Dis. 2019 Oct 14;18:177. doi: 10.1186/s12944-019-1125-1 (PMC6792215; doi:10.1186/s12944-019-1125-1)

**Additional file 1: Table S1.** Primer sequences used for qPCR.

| **Gene name** | **Primer sequence 5’-3’** | **Method** |
| --- | --- | --- |
| **SREBP-1** | F: TGCATTTTCTGACACGCTTC  R: CCAAGCTGTACAGGCTCTCC | qPCR |
| **SREBP-2** | F: TGGCTTCTCTCCCTACTCCA  R: GAGAGGCACAGGAAGGTGAG | qPCR |
| **GAPDH** | F: GAAGGTGAAGGTCGGAGTC  R: GAAGATGGTGATGGGATTTC | qPCR |

**Table S2.** Pearson/Spearman correlation coefficients between circulating leukocyte SREBP-1, SREBP-2 and CAD risk factors in controls.

|  |  | **SREBP-1** | | | | |  | **SREBP-2** | | | | |
| --- | --- | --- | --- | --- | --- | --- | --- | --- | --- | --- | --- | --- |
|  |  | **Control (n=198)** | |  | **CAD (n=218)** | |  | **Control (n=198)** | |  | **CAD (n=218)** | |
|  |  | r | *P* |  | r | *P* |  | r | *P* |  | r | *P* |
| **TC** |  | 0.257 | 0.001 |  | 0.059 | 0.384 |  | -0.026 | 0.726 |  | 0.068 | 0.321 |
| **TG** |  | 0.038 | 0.616 |  | 0.091 | 0.181 |  | -0.056 | 0.128 |  | -0.025 | 0.718 |
| **HDL-C** |  | 0.145 | 0.053 |  | 0.171 | 0.011 |  | 0.007 | 0.929 |  | 0.054 | 0.941 |
| **LDL-C** |  | 0.162 | 0.030 |  | 0.013 | 0.853 |  | -0.039 | 0.603 |  | 0.060 | 0.381 |
| **Apo-A1** |  | 0.296 | <0.001 |  | 0.145 | 0.033 |  | -0.092 | 0.222 |  | 0.029 | 0.666 |
| **Apo-B** |  | 0.232 | 0.002 |  | -0.061 | 0.370 |  | -0.097 | 0.199 |  | 0.044 | 0.522 |
| **LP(a)** |  | 0.055 | 0.464 |  | 0.091 | 0.179 |  | 0.029 | 0.702 |  | 0.071 | 0.295 |
| **AST** |  | 0.062 | 0.411 |  | -0.088 | 0.197 |  | -0.061 | 0.417 |  | -0.169 | 0.052 |
| **CK-MB** |  | -0.170 | 0.023 |  | -0.201 | 0.003 |  | 0.031 | 0.681 |  | -0.016 | 0.814 |
| **LDH** |  | 0.038 | 0.615 |  | -0.146 | 0.031 |  | -0.122 | 0.104 |  | -0.079 | 0.243 |
| **HBDH** |  | -0.068 | 0.369 |  | -0.123 | 0.069 |  | -0.125 | 0.096 |  | -0.078 | 0.254 |
| **hs-CRP** |  | -0.284 | <0.001 |  | -0.165 | 0.015 |  | -0.006 | 0.939 |  | 0.028 | 0.679 |
| **HCY** |  | 0.038 | 0.615 |  | -0.286 | <0.001 |  | 0.073 | 0.331 |  | 0.080 | 0.238 |
| **CysC** |  | 0.380 | <0.001 |  | 0.150 | 0.027 |  | -0.132 | 0.079 |  | 0.091 | 0.180 |
| **GSP** |  | 0.010 | 0.894 |  | 0.229 | 0.001 |  | -0.015 | 0.178 |  | 0.039 | 0.566 |
| **SREBP-2** |  | 0.373 | <0.001 |  | 0.512 | <0.001 |  | / | / |  | / | / |

**Table S3.** Baseline and clinical characteristics of the participants.

| **Characteristic** | **Controls**  **(n=73)** | **CAD patients**  **(n=67)** | ***p* value** |
| --- | --- | --- | --- |
| **Demographics** |  |  |  |
| Age (years) | 58.36±6.58 | 56.72±7.91 | 0.187 |
| Male | 50 | 46 | 0.983 |
| **Risk factors** |  |  |  |
| Hypertension (%) | 2 | 38 | <0.001 |
| Diabetes mellitus (%) | 1 | 16 | <0.001 |
| hyperlipidemia (%) | 0 | 9 | 0.001 |
| **Clinical parameters** | | | |
| TC (mmol/L) | 4.98±0.85 | 3.88±1.32 | <0.001 |
| TG (mmol/L) | 1.11(0.86, 1.43) | 1.21 (0.82, 2.02) | 0.229 |
| HDL-C (mmol/L) | 1.41±0.34 | 1.02±0.33 | <0.001 |
| LDL-C (mmol/L) | 2.42±0.57 | 2.00±0.85 | 0.001 |
| Apo-A1 (g/L) | 1.61±0.21 | 1.45±0.35 | 0.001 |
| Apo-B (g/L) | 0.94±0.17 | 0.84±0.27 | 0.008 |
| LP(a) (mg/L) | 41.91 (18.00, 126.57) | 84.19 (31.73, 215.03) | 0.019 |
| AST (IU/L) | 24.00 (21.00, 28.00) | 25.00 (18.00,35.00) | 0.670 |
| CK-MB (IU/L) | 8.90±6.75 | 21.09±8.32 | <0.001 |
| LDH (IU/L) | 194.30±33.21 | 196.76±72.45 | 0.800 |
| HBDH (IU/L) | 121.92±25.51 | 130.21±57.77 | 0.282 |
| hs-CRP (mg/L) | 3.57 (2.13, 5.97) | 7.11 (5.38, 12.55) | <0.001 |
| HCY (μmol/L) | 10.40 (9.40,12.70) | 12.20 (9.50, 14.00) | 0.038 |
| CysC (mg/L) | 0.59±0.13 | 0.52±0.22 | 0.017 |
| GSP (mmol/L) | 2.63(2.48, 2.74) | 2.65(2.39, 2.89) | 0.814 |
| **SREBP-1 (fold change)** | 1.00±1.04 | 0.58±0.39 | <0.001 |
| **SREBP-2 (fold change)** | 1.00±0.92 | 0.76±0.87 | 0.124 |
| **miR-33a*(fold change)** | 1.00±2.92 | 0.86±2.82 | 0.767 |
| **miR-33b*(fold change)** | 1.00±2.42 | 1.09±2.16 | 0.866 |

CAD, coronary artery disease; TC, total cholesterol; TG, Triglycerides; HDL-C, high-density lipoprotein cholesterol; LDL-C, low-density lipoprotein cholesterol; Apo-A1, apolipoprotein A1; Apo-B, apolipoprotein B; LP(a), lipoprotein a; AST, Aspartate aminotransferase; CK-MB, creatine kinase; LDH, lactate dehydrogenase; HBDH, hydroxybutyrate dehydrogenase; Hs-CRP, high-sensitivity C-reactive protein; HCY, homocysteine; CysC, Cystatin C; glycated serum protein (GSP).

Values represent mean ± SD and median (interquartile range) for continuous variables and n (%) for categorical variables; p values for comparison between groups using the Student’s t-test for parametric data or the corresponding Mann-Whitney U test for nonparametric continuous variables, and the χ^2^ test for categorical variables.

**Table S4.** Pearson/Spearman correlation coefficients between plasma mir-33 and CAD risk factors in all participants tested for miR-33.

|  |  | **miR-33a** | |  | **miR-33b** | |
| --- | --- | --- | --- | --- | --- | --- |
|  |  | **（n=140）** | |  | **（n=140）** | |
|  |  | **r** | ***P*** |  | **r** | ***P*** |
| **TC** |  | 0.037 | 0.669 |  | -0.023 | 0.821 |
| **TG** |  | 0.169 | 0.049 |  | 0.079 | 0.438 |
| **HDL-C** |  | 0.004 | 0.963 |  | -0.100 | 0.327 |
| **LDL-C** |  | 0.070 | 0.416 |  | 0.047 | 0.644 |
| **Apo-A1** |  | 0.039 | 0.654 |  | -0.135 | 0.187 |
| **Apo-B** |  | 0.062 | 0.476 |  | 0.032 | 0.755 |
| **LP(a)** |  | -0.121 | 0.159 |  | -0.105 | 0.304 |
| **AST** |  | 0.217 | 0.011 |  | 0.005 | 0.960 |
| **CK-MB** |  | 0.108 | 0.209 |  | -0.020 | 0.848 |
| **LDH** |  | -0.045 | 0.601 |  | 0.055 | 0.594 |
| **HBDH** |  | -0.043 | 0.617 |  | 0.102 | 0.316 |
| **hs-CRP** |  | 0.011 | 0.902 |  | -0.063 | 0.537 |
| **HCY** |  | -0.102 | 0.236 |  | 0.067 | 0.511 |
| **CysC** |  | 0.059 | 0.497 |  | 0.014 | 0.891 |
| **GSP** |  | 0.240 | 0.005 |  | -0.003 | 0.976 |
| **SREBP-1** |  | -0.079 | 0.358 |  | 0.192 | 0.058 |
| **SREBP-2** |  | -0.035 | 0.689 |  | 0.168 | 0.099 |

The Pearson correlation coefficient was selected to measure the strength of the liner relationship between normally-distributed variables and the Spearman correlation coefficient was used to evaluate non-normally distributed data.

**Table S5.** Clinical characteristics and SREBP mRNA in CAD with different chronic pathological conditions.

| **Characteristic** | **CAD patients with*** | | |
| --- | --- | --- | --- |
|  | **Hypertension**  n=125 | **Hyperlipidemia**  n=32 | **Diabetes**  n=46 |
| **Demographics** |  |  |  |
| **Age (years)** | 59.75±8.67 | 55.62±7.28 | 61.54±8.30 |
| **Male** | 74 | 19 | 32 |
| **Clinical parameters** | | | |
| **TC (mmol/L)** | 3.71±1.11 | 4.27±0.98 | 3.75±1.26 |
| **TG (mmol/L)** | 1.31(0.82, 2.06) | 2.05(1.31, 2.94) | 1.48(0.85, 2.70) |
| **HDL-C (mmol/L)** | 1.00±0.25 | 1.00±0.26 | 0.94±0.25 |
| **LDL-C (mmol/L)** | 1.92±0.74 | 2.31±0.66 | 2.00±0.83 |
| **Apo-A1 (g/L)** | 1.47±0.27 | 1.57±0.27 | 1.48±0.30 |
| **Apo-B (g/L)** | 0.82±0.23 | 0.97±0.21 | 0.85±0.26 |
| **LP(a) (mg/L)** | 124.30±106.48 | 129.66±116.80 | 121.04±116.42 |
| **AST (IU/L)** | 24.00(18.00, 37.00) | 28.00(18.00, 44.50) | 22.50(18.00, 37.50) |
| **CK-MB (IU/L)** | 16.06±9.62 | 15.09±9.74 | 15.45±10.02 |
| **LDH (IU/L)** | 199.57±58.49 | 181.47±42.27 | 188.26±59.39 |
| **HBDH (IU/L)** | 130.36±44.50 | 116.25±29.27 | 123.26±46.17 |
| **hs-CRP (mg/L)** | 7.77(4.99, 12.69) | 8.17(4.81, 15.81) | 8.44(5.53, 13.73) |
| **HCY (μmol/L)** | 11.80(8.60,13.75) | 10.70(7.45, 13.65) | 10.70(6.50, 13.02) |
| **CysC (mg/L)** | 0.61±0.24 | 0.60±0.30 | 0.67±0.27 |
| **GSP (mmol/L)** | 2.70(2.43, 2.97) | 2.71(2.54, 2.90) | 2.98(2.77, 3.78) |
| **SREBP-1** | 0.48±0.78 | 0.70±1.09 | 0.71±1.11 |
| **SREBP-2** | 0.86±1.25 | 1.03±1.12 | 1.01±1.64 |
| **Pharmacological treatments (n)** | | | |
| **Anti-hypertension** | 119 | 11 | 21 |
| **Anti-diabetic** | 24 | 15 | 45 |
| **Statins** | 74 | 22 | 25 |

*** An individual might be suffered more than one primary disorder.**

Fig S1. The flow charts of the study. A, flow chart of case-control trials; B, flow chart of the *in vitro* study


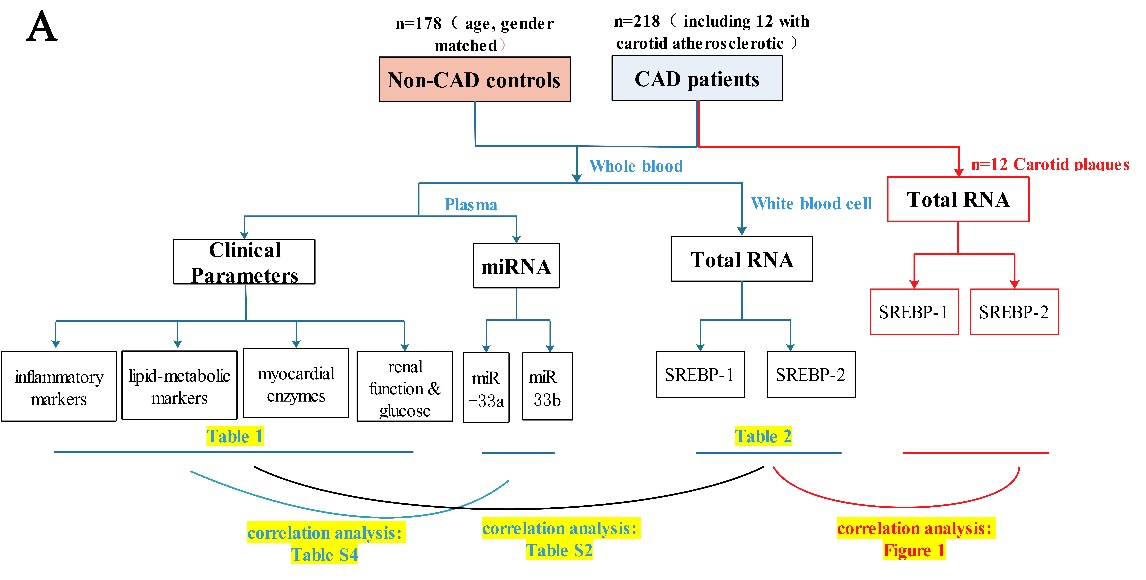


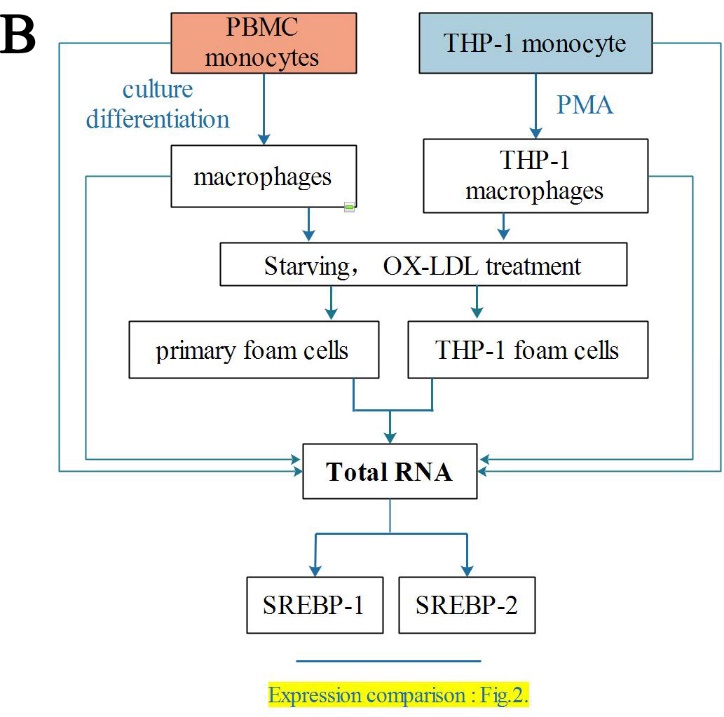


Fig S2. The flow charts of sample size calculation.


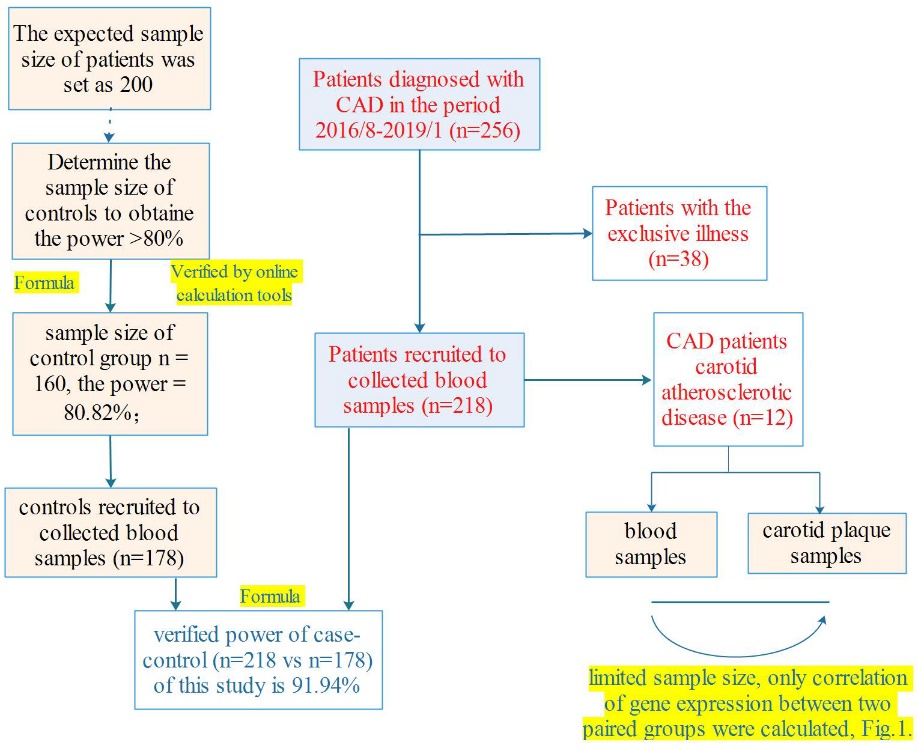


**Calculate Sample Size to Compare 2 Means: 2-Sample, 2-Sided**

Suppose the two groups are 'A (controls)' and 'B (CAD patients)'. We perform a two-sample test to determine whether the mean in group A, *μ_A_*, is different from the mean in group B, *μ_B_*.

The hypotheses are

*H*_0_: *μ_A_*−*μ_B_*=0

*H*_1_: *μ_A_*−*μ_B_*≠0

where the ratio between the sample sizes of the two groups is

*κ*=*n_A_/n_B_*

Formulas

we used the following formulas to calculate sample size and power, respectively:

*n_A_* = *κn_B_* and ${nB=\left( 1+\frac{1}{\kappa} \right)\left( \sigma\frac{z1-\frac{\alpha}{2}+z1-\beta}{\kappa\mu A-\mu B} \right)}^{2}$

1−*β* = Φ(*z*−*z*_1−_*_α_*_/2_) + Φ(−*z*−*z*_1−_*_α_*_/2_), *z*$=\left( \frac{\mu A-\mu B}{\sigma\sqrt{\frac{1}{nA}+\frac{1}{nB}}} \right)$

where

- *κ*=*n_A_*/*n_B_* is the matching ratio,
- *σ* is standard deviation
- *μ_A_* is the estimated Group“A” mean
- *μ_B_* is the estimated Group “B” mean
- α is Type I error
- β is Type II error, meaning 1−β is power

Fig S3. 12 Carotid plaques from CAD patients undergoing carotid endarterectomy


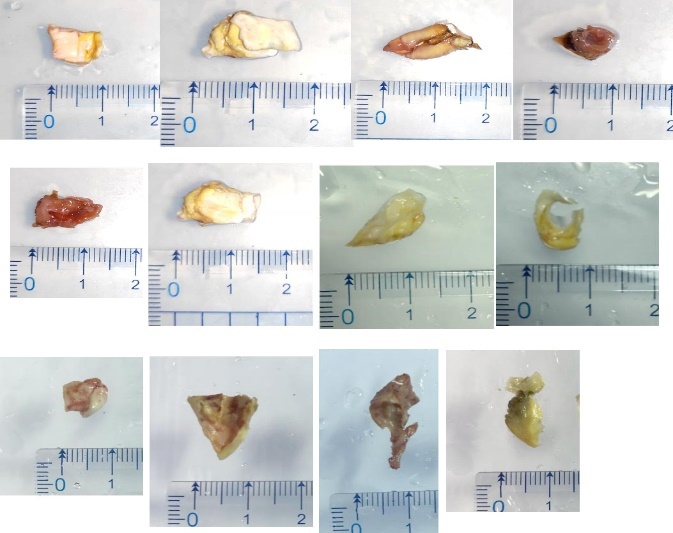

Supplement: Supplementary file 1 — Additional file 1: Table S1. Primer sequences used for qPCR. Table S2. Pearson/Spearman correlation coefficients between circulating leukocyte SREBP-1, SREBP-2 and CAD risk factors in controls. Table S3. Baseline and clinical characteristics of the participants. Table S4. Pearson/Spearman correlation coefficients between plasma mir-33 and CAD risk factors in all participants tested for miR-33. Table S5. Clinical characteristics and SREBP mRNA in CAD with different chronic pathological conditions. Figure S1. The flow charts of the study. A, flow chart of case-control trials; B, flow chart of the in vitro study. Figure S2. The flow charts of sample size calculation. Figure S3. 12 Carotid plaques from CAD patients undergoing carotid endarterectomy. [file 12944_2019_1125_MOESM1_ESM.docx]
